# Characteristics of Osteoporosis & Osteoporotic Fractures in Korea Based on Health Insurance Review and Assessment (HIRA) Database: 2009–2017

**DOI:** 10.3390/healthcare9030324

**Published:** 2021-03-14

**Authors:** Ho-Seok Oh, Sung-Kyu Kim, Hyoung-Yeon Seo

**Affiliations:** Department of Orthopaedic Surgery, Chonnam National University Medical School and Hospital, Gwangju 61469, Korea; koreankid07@naver.com (H.-S.O.); hyseo2001@daum.net (H.-Y.S.)

**Keywords:** osteoporosis, osteoporotic fractures, Korea, insurance, big data, epidemiology

## Abstract

To investigate the incidence and characteristics of osteoporosis and osteoporotic fractures in Korea, we used the Health Insurance Review and Assessment Service (HIRA) database. Patients over 50 years old, who were diagnosed or treated for osteoporosis and osteoporotic fractures in all hospitals and clinics, were analyzed between 1 January 2009 and 31 December 2017 by using the HIRA database that contains prescription data and diagnostic codes. These data were retrospectively analyzed by decade and age-specific and gender-specific incidents in each year. We also evaluated other characteristics of patients including medication state of osteoporosis, primary used medical institution, regional-specific incidence of osteoporosis, and incidence of site-specific osteoporotic fractures. The number of osteoporosis patients over 50 years old, as diagnosed by a doctor, steadily increased from 2009 to 2017. The number of osteoporosis patients was notably greatest in the 60′s and 70′s age groups in every study period. Patients undergoing treatment for osteoporosis increased significantly (96%) from 2009 to 2017. Among the patients diagnosed with osteoporosis, the proportion who experienced osteoporotic fracture increased gradually (60%) from 2009 to 2017. The number of patients with osteoporotic fractures of the spine and hip was highest in the 70 to 90 age range, and the number of patients with osteoporotic fractures in the upper and lower extremities was highest in the 50 to 70 age range. Understanding the trends of osteoporosis in Korea will contribute to manage the increased number of patients with osteoporosis and osteoporotic fractures.

## 1. Introduction

Osteoporosis is a disease characterized by low bone mass and microstructural deterioration of bone tissue. This is associated with bone fragility and increases the risk of fracture [1,2,3]. Recently, the number of patients with osteoporosis has increased rapidly as population aging is ongoing around the world [4,5,6,7,8]. As a result, the cost and implications of osteoporotic fractures for national health care systems are also rapidly increasing [9,10].

Korea (Republic of Korea, South Korea) is one of the fastest aging societies, and the Korean population over 50 years old will grow to 57% in 2050 [4,11]. This indicates that the proportion of the population at risk for osteoporosis and osteoporotic fracture in Korea will increase [4]. Osteoporotic fractures, especially spine or hip fractures, are one of the main causes of morbidity and disability in elderly patients. This increases the socioeconomic burden on the health care system [9,12,13]. To manage the socioeconomic burden of osteoporosis and osteoporotic fractures, basic epidemiologic data, such as incidence and characteristics, should be examined [1,14,15,16]. Although some epidemiological studies have been conducted on the nation-wide incidence of osteoporosis and osteoporotic fractures in Korea, few epidemiologic studies on the characteristics of osteoporosis, such as the current state of medical service, primary medical institution used, regional-specific incidence of osteoporosis and incidence of site-specific osteoporotic fractures, have been conducted. In addition, this study is the most recent incidence research on osteoporosis in Korea.

In Korea, the statutory national health insurance system is run by the government with a central database called the Health Insurance Review and Assessment Service (HIRA). This database contains all of the prescription and treatment claim records for more than 99% of the Korean population [17]. The aim of this study is to investigate the incidence and characteristics of osteoporosis and osteoporotic fractures in Korea by using the HIRA database in order to manage the increased number of patients with osteoporosis and osteoporotic fractures.

## 2. Materials and Methods

Patients greater than 50 years old who were diagnosed or treated with osteoporosis and osteoporotic fractures in all hospitals and clinics between 1 January 2009 and 31 December 2017 were analyzed. In the HIRA database, only cases which incurred medical expenses by a patient visiting a doctor are recorded. This means that an osteoporosis patient identified through the HIRA database is a case in which the doctor considers various diagnostic factors comprehensively and treats it as an osteoporosis patient. In order to derive more accurate results, we tried to establish a clear operational definition for osteoporosis and osteoporotic fracture. 

The operational definition for this study include the use of exclusive medications for osteoporosis treatment—bisphosphonate, estrogen, tissue selective estrogen complex (TSEC), selective estrogen receptor modifier (SERM), receptor activators of nuclear factor kappa-B ligand (RANKL) inhibitor, parathyroid hormone (PTH) and calcitriol. We used the diagnostic code for osteoporosis (ICD-10 codes M80-82 for osteoporosis) and codes for osteoporotic fracture (M48.4, M48.5, S22.0, S22.1, S32.0 for spine fracture, S42.2, S42.3 for proximal humerus fracture, S52.5, S52.6 for distal radius fracture and S72.0, S72.1 for hip fracture for patients over 50 years old) to analyze the characteristics of osteoporosis and osteoporotic fracture in Korea [1]. To avoid statistical duplication, we limited the case for outpatients only with at least two ICD-10 codes for osteoporosis diagnosis to 12 months, and for inpatients those who stayed more than 2 days [4]. These data were retrospectively evaluated to determine the age- and gender-specific incidence for each year. 

To identify trends in the incidence of osteoporotic fractures in osteoporotic patients, osteoporotic fractures were categorized by location, which included spine, upper extremity (proximal humerus, distal radius) and hip. The incidence of each group was identified for each year and evaluated to determine the annual age- and gender-specific incidence for each year.

The HIRA database also includes the type of medical institution (orthopedic surgery, internal medicine, obstetrics and gynecology, neurosurgery, family medicine, etc.) where osteoporosis patients are diagnosed and treated, and the proportion of patients who are diagnosed and treated in urban and rural settings each year. 

## 3. Results

**Figure 1:**
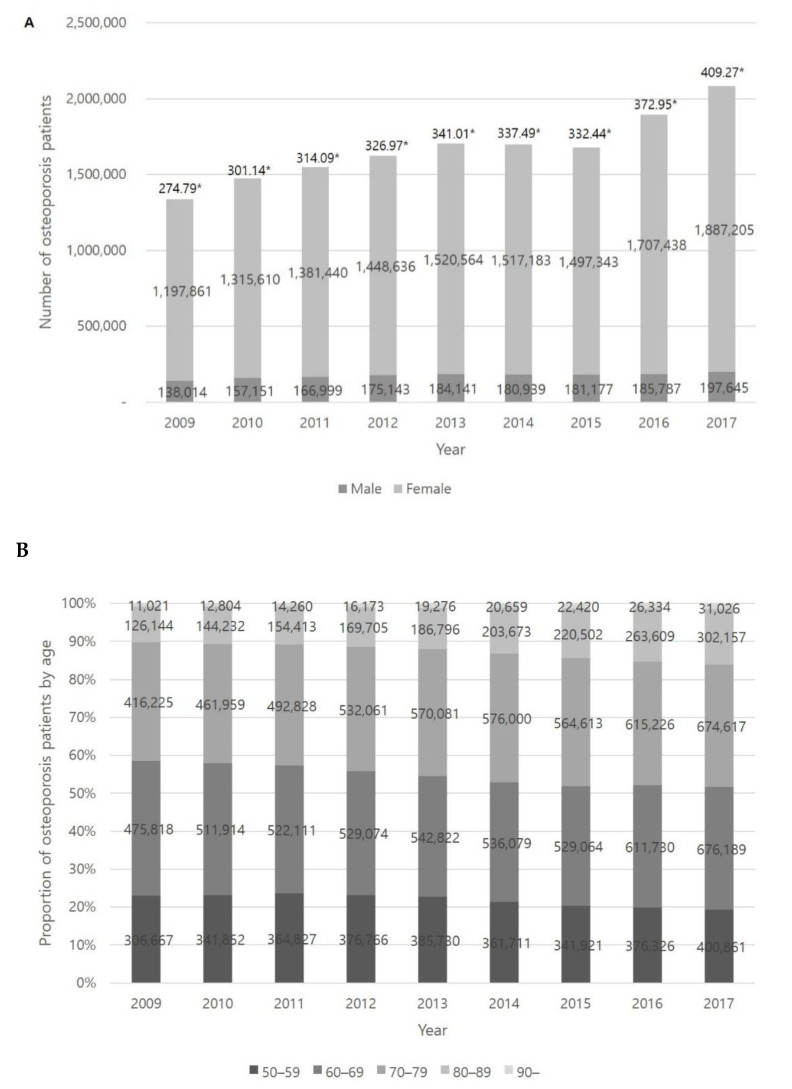
The number of osteoporosis patients in Korea according to gender (**A**) and age (**B**) each year. * Incidence rate of osteoporosis patients (n/10,000).

The number of osteoporosis patients over 50 years old diagnosed by a doctor steadily increased over the study period. From 2009 to 2017, the incidence rate of osteoporosis patients increased 274.79/10,000 to 409.27/10,000. The number of osteoporosis patients increased by 58% in women (from 1,197,861 to 1,887,205) and 43% in men (from 138,014 to 197,645). Among this population, approximately 90% were female patients in each of the study periods (Figure 1A). In all age groups, the number of osteoporosis patients in their 60s and 70s was the greatest. It is notable that the proportion of osteoporosis patients in their 80s continuously increased from 9% (126,144) in 2009 to 14% (302,157) in 2017 and this change was greatest among all ages. The incidence of osteoporosis in each decade showed a similar pattern during each of the study periods (Figure 1B). 

**Figure 2:**
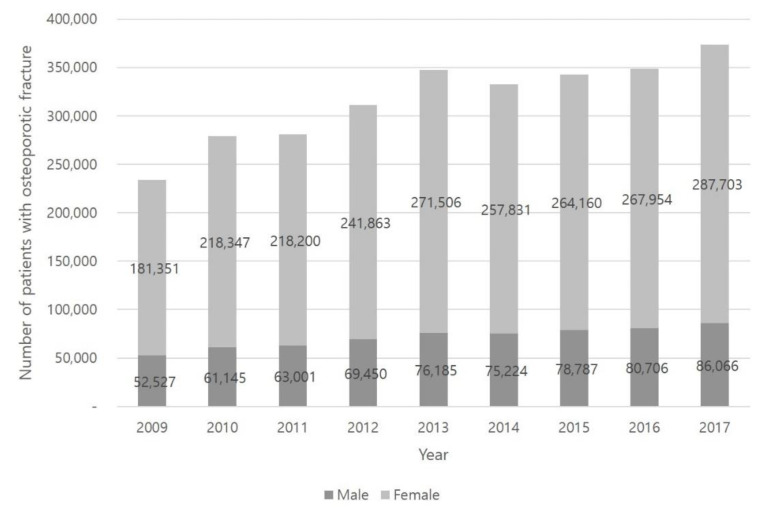
The number of patients with osteoporotic fracture each year.

**Figure 3:**
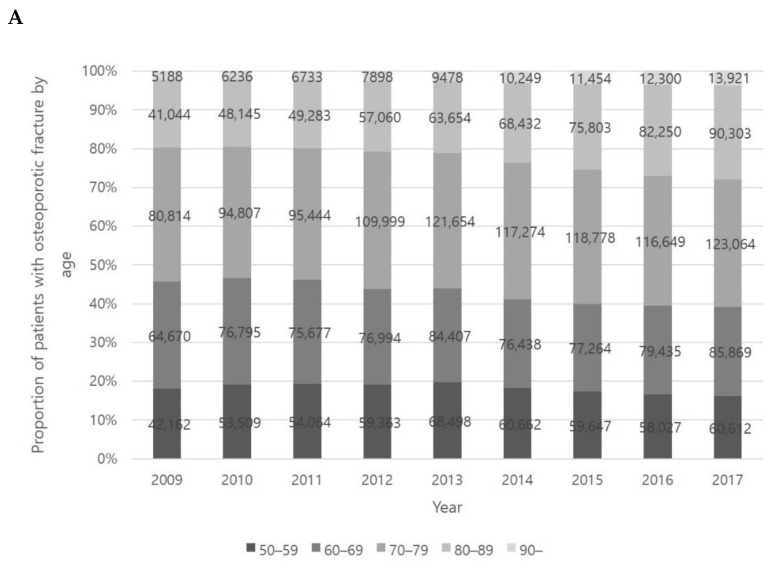

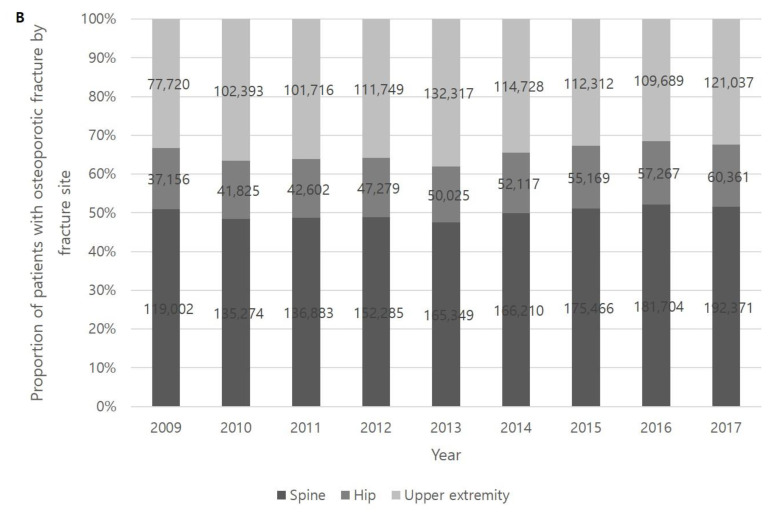
Age-specific incidence of osteoporotic fracture (**A**) and incidence of osteoporotic fracture according to fracture site (**B**).

The number of osteoporotic fracture patients gradually increased, increasing by 60% from 2009 to 2017 (from 233,878 to 373,769). Patients with osteoporotic fractures maintained a similar proportion of the total population at 18% in 2009 and 2017 (Figure 2). We found that the number of patients with osteoporotic fractures was also highest in the 60s and 70s age groups. The proportion of osteoporotic fractures in the 80s age group increased (18% in 2009 and 24% in 2017) as well (Figure 3A).

Throughout the study period, the most common location of osteoporotic fractures was the spine, followed by the upper extremity and hip. The number of osteoporotic fracture patients over 50 years of age increased steadily at all locations including spine, hip and upper extremity (Figure 3B). 

**Figure 4:**
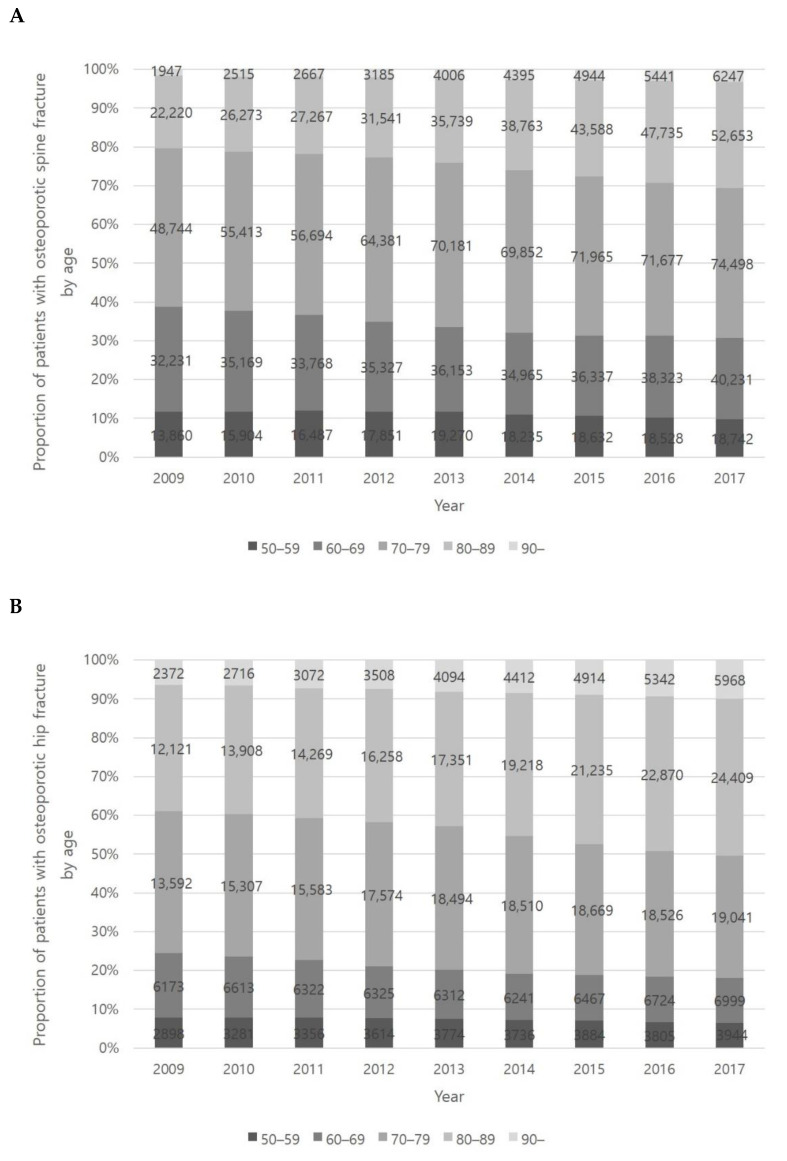

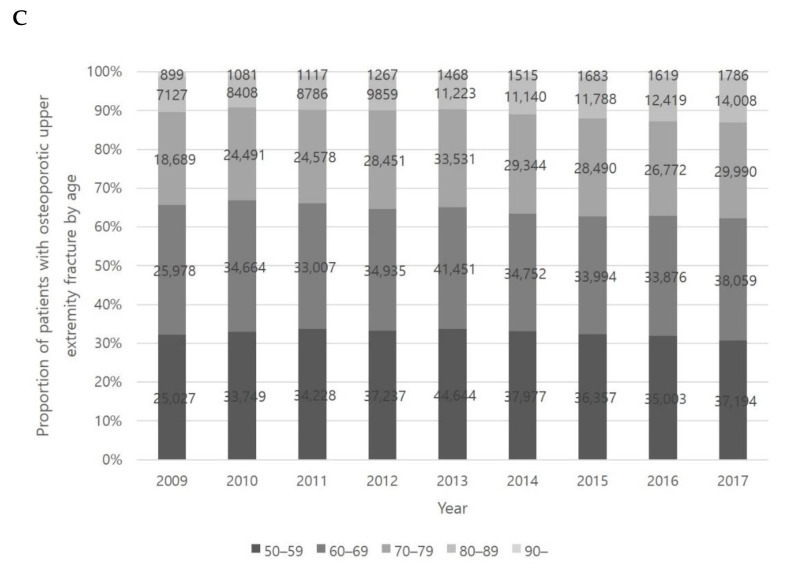
Incidence of osteoporotic spine fracture (**A**), hip fracture (**B**), and upper extremity fracture (**C**).

The number of patients with spine osteoporotic fractures tended to be highest in the 70 to 90 age range (approximately 41% in the 70s and 23% in the 80s). Characteristically, the proportion of spine osteoporotic fractures in the 80s group increased steadily during the study period (19% in 2009 and 27% in 2017) (Figure 4A). The incidence of hip osteoporotic fractures shows a similar trend with spine osteoporotic fractures. Hip osteoporotic fractures also occurred more frequently in the 70 and 90 age range (approximately 35% in the 70s and 36% in the 80s) and showed a steady increase in the 80s age group (33% in 2009 and 40% in 2017) (Figure 4B). The number of patients with upper extremity osteoporotic fractures was highest in the 50 to 70 age group (approximately 33% in the 50s and 32% in the 60s). In the case of upper extremity osteoporotic fractures, the proportions of each age group remain similar during the study period (Figure 4C).

**Figure 5:**
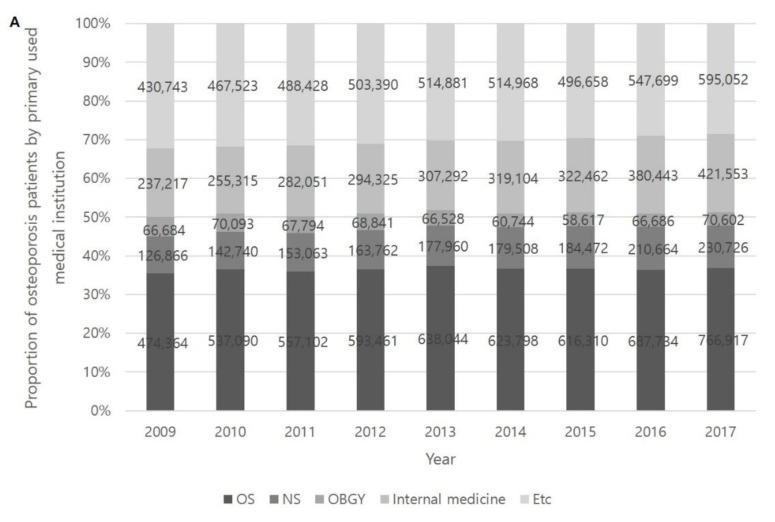

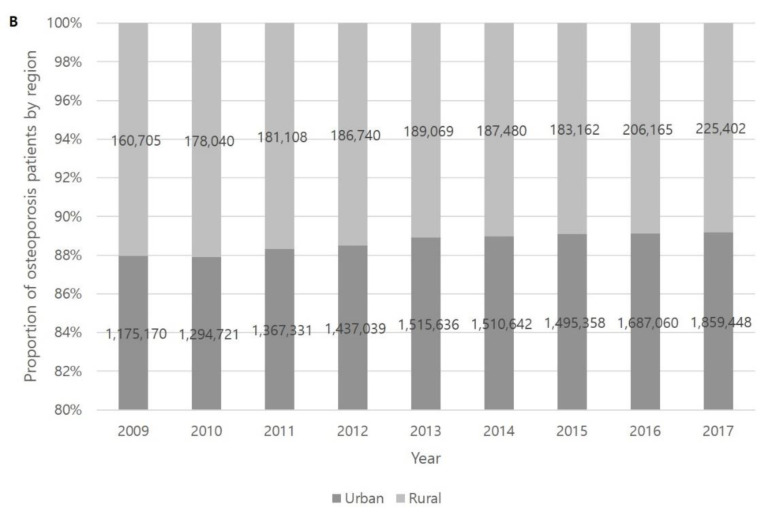
Distribution of primary treatment institutions in osteoporosis patients (**A**) and the number of osteoporosis patients in urban and rural settings (**B**). OS: orthopedic surgery; NS: neurosurgery; OBGY: obstetrics and gynecology. Note: The *Y*-axis of the graph starts at 80%.

Patients diagnosed with osteoporosis were mostly treated in an orthopedic surgery department, followed by international medicine, neurosurgery and obstetrics and gynecology departments (Figure 5A). The proportion of departments providing treatment did not show a significant difference during the study period. Urban people (approximately 89%) tended to be diagnosed with osteoporosis more often than rural people (approximately 11%) (Figure 5B). 

## 4. Discussion

As the social cost of osteoporosis has increased, the Korean government and medical officials have recently highlighted the importance of osteoporosis. There were few studies conducted that investigated the prevalence of osteoporosis in Korea using the HIRA database [1,4,16]. Sunmee Jang et al. [1] studied epidemiology of osteoporosis in Korea using the HIRA database in 2007. There were about 1,230,580 patients identified as osteoporosis-diagnosed patients aged over 45. Compared to their study, although the age criteria and year were different, our result on the number of osteoporosis-diagnosed patients was 1,335,875 in 2009. Because the HIRA database does not include the measurement of bone mineral density (BMD) using dual energy X-ray absorptiometry (DXA), it is very important to establish an operational definition of osteoporosis to analyze the HIRA database more accurately. Further study is needed because it is not possible to know which criteria are more accurate at present. However, this study is meaningful since it provides the latest epidemiology of osteoporosis and osteoporotic fracture, and also presents the characteristics of osteoporosis-diagnosed patients and medical service utilization in Korea.

In our study, there was a steady increase in osteoporotic-diagnosed patients from 2009 to 2017, and the increasing trend in osteoporosis is probably the result of increase in longevity and changes in lifestyles. Recently, many organizations focused on osteoporosis have provided various educational programs for clinicians in Korea. These programs have greatly increased doctor’s awareness of osteoporosis and treatments. Female osteoporosis patients between the age of 60 and 80 years make up the majority of the entire adult population, which means that a the prevalence of osteoporosis in postmenopausal women is high, and a huge social burden of postmenopausal osteoporosis occurs. This result highlights the need for active osteoporosis screening among postmenopausal women [18].

Osteoporotic fractures have become a public health problem because, in many cases, surgery is required. In addition, osteoporotic fractures cause many other problems in patients including a long time returning to daily life after fracture and a higher mortality rate as compared to the general population [18,19]. Our study revealed an increasing pattern of fracture incidence and a changing trend in the location of osteoporotic fractures. Upper extremity osteoporotic fractures show a particularly different pattern of occurrence than hip and vertebral fractures. Our study showed that upper extremity osteoporotic fracture is a significant part of the total osteoporotic fractures in relatively younger patients. This finding may be associated with neuromuscular reflexes that change with age, so older individuals tend not to fall over with outstretched arms, but to the side or back [20].

Urban people tend to be diagnosed with osteoporosis more often than rural people according to our study. This result suggests that urban people have better access to medical care than rural people. However, the urban and rural total populations were not accurately surveyed in our study, so they were expressed as absolute numbers for patients with osteoporosis. Further studies are needed to compare the relative proportions of each of the urban and rural datasets to determine which areas are more susceptible to osteoporosis.

There were some limitations to the study using an insurance claim database. The insurance claim database failed to include prescriptions outside insurance coverage (less than 1% in Korea), and there may be incorrect diagnostic coding and misclassification errors. Since the database from HIRA does not contain BMD measurement using DXA, developing the operational definition of osteoporosis to identify patients with osteoporosis using diagnostic codes is necessary. 

## 5. Conclusions

The results of our study will provide latest information including the characteristics of patients with osteoporosis and osteoporotic fractures in Korea. Understanding the trends of osteoporosis in Korea will contribute to managing the increased number of patients with osteoporosis and osteoporotic fractures.

## Data Availability

The data presented in this study are available on request from the corresponding author.
